# The contributions of executive functions to mathematical learning difficulties and mathematical talent during adolescence

**DOI:** 10.1371/journal.pone.0209267

**Published:** 2018-12-13

**Authors:** Roberto A. Abreu-Mendoza, Yaira Chamorro, Mauricio A. Garcia-Barrera, Esmeralda Matute

**Affiliations:** 1 Instituto de Neurociencias, CUCBA, Universidad de Guadalajara, Guadalajara, Jalisco, México; 2 Rutgers University, Newark, New Jersey, United States of America; 3 Department of Psychology, University of Victoria, Victoria, British Columbia, Canada; French National Center for Scientific Research (CNRS) & University of Lyon, FRANCE

## Abstract

Are mathematical learning difficulties caused by impairment of the abilities that underlie mathematical talent? Or are mathematical difficulties and talent qualitatively different? The main goal of this study was to determine whether mathematical learning difficulties are explained by the same executive functions as mathematical talent. We screened a pool of 2,682 first-year high school students and selected 48 for evaluation, dividing them into three groups: those with mathematical learning difficulties (n = 16), those with typical performance (n = 16), and those with mathematical talent (n = 16). Adolescents from the learning difficulties and talented groups had age, reading skills, and verbal and non-verbal intelligence that were similar to those of the typical performance group. Participants were administered a suite of tasks to evaluate verbal and visual short-term memory and executive functions of inhibition, shifting, and updating. Different executive functions showed different contributions at the two ends of the math ability continuum: lower levels of performance in updating visual information were related to mathematical learning difficulties, while greater shifting abilities were related to mathematical talent. Effect sizes for the differences in performance between groups were large (Hedges' *g* > 0.8). These results suggest that different executive functions are associated with mathematical learning difficulties and mathematical talent. We discuss how these differences in executive functions could be related to the different types of mathematical abilities that distinguish the three groups.

## Introduction

During adolescence, students are expected to acquire competency in a range of mathematical subjects, such as algebra, trigonometry, and calculus. To do so, they must build their knowledge of new concepts on a previously learned foundation of arithmetic with natural and rational numbers. Mathematical attainment will have an impact on various aspects of their adult lives, including employment opportunities [1,2], health [3], and socioeconomic status [4]. At the beginning of high school, however, there are large individual differences in mathematical skills. Some adolescents struggle still with basic arithmetic, while others are prepared to build the mathematical knowledge needed to pursue a science, technology, engineering, and math (STEM) career.

What are the cognitive abilities associated with these individual differences in mathematical skills? Executive functions, the abilities to form, plan, and carry out goal-directed behaviors [5], have been identified as playing a key role in explaining such differences throughout development [6–8]. Impaired executive functions, especially working memory, have also been proposed as a core deficit in individuals with mathematical learning difficulties (MLD). However, only recently have studies begun to report the contribution of different executive functions to mathematical talent. The current study aimed to investigate whether the same executive functions contribute to the whole continuum of mathematical aptitude, or whether specific functions contribute to MLD or mathematical talent.

### Mathematical aptitude in adolescence

By the end of middle school, adolescents in Mexico should have developed a solid knowledge of the arithmetic of natural and rational numbers and should have started to develop their understanding of basic concepts of algebra and statistics [9]. However, first-year high school students still appear to lag behind these expectations [10,11]. Recently, Abreu-Mendoza, Chamorro, and Matute [10] reported that first-year Mexican high school students still have difficulties in solving arithmetic problems that involve fractions and decimals. Using a large sample (*N* = 1236), they performed an exploratory factor analysis on the 40 items of the Math Computation subtest of the Wide Range Achievement Test-4 (WRAT-4) [12]. A solution was identified with three factors: the first was the addition, subtraction, multiplication, and division of multi-digit numbers with and without decimals (arithmetic); the second was the solution of arithmetic problems of like fractions and simple equations (fractions and basic algebra); and the third was the conversion of decimals to percentages or fractions, and the solution of arithmetic problems involving fractions with different denominators (rational numbers). Although these abilities should have been consolidated at this stage of education, none of these factors showed a percentage of correct responses above 62%. Such difficulties are not exclusive to Mexican students. At roughly the same age, only a small percentage (27%) of U.S. eighth graders correctly estimate the sum of 12/13 + 7/8 [13]. Taken together, these studies suggest that individual differences in mathematical abilities during adolescence are found mostly in arithmetic problems that involve fractions and decimals.

### Executive functions in adolescence

The executive functions are general purpose mechanisms that modulate the operation of various cognitive subprocesses [14] and allow an organized, purposeful, and goal-directed behavior [15]. Various models have described the abilities that fall under the executive function umbrella term. Miyake et al. [14] proposed "shifting," "inhibition," and "updating" as three basic, separated, yet related executive components that can be accurately operationalized and that are implicated in the performance of more complex executive behaviors. Shifting concerns the ability to switch from one mindset to another, often according to rules that would be incompatible with the other [16]. Updating involves monitoring and coding incoming information for relevance to a given task [17] and is closely related to the concept of working memory. Inhibition is the ability to deliberately suppress dominant, automatic, or prepotent responses when necessary [14].

Many studies have adopted this multifactorial framework. However, this model of executive functions is based on adult data, and it is not clear if it also describes developmental stages [18], especially when executive functions follow a protracted developmental path from childhood to adulthood [19]. In this regard, Lee et al. [18] reported that a two-factor model (combining inhibitory control and switching measures) provides a better fit during childhood (six to ten years of age) than the three-factor structure. The former is feasible until 11 years of age, and the three executive components became more differentiated around 15 years of age. A recent systematic review and re-analysis of confirmatory factor analysis-based models demonstrated a higher acceptance of parsimonious models (1 or 2 factors) in childhood and early adolescence [20]. Thus, late adolescence seems to be a good developmental stage to assess the three components proposed by Miyake and colleagues [14,21] in relation to academic achievement.

### Mathematical aptitude and executive functions

Numerous studies have shown the importance of executive functions to the acquisition of mathematical skills. Performance in executive function tasks is associated with individual differences in numerical abilities from early childhood [7,8,22], and through childhood [23–25], adolescence, and adulthood [26–28].

Executive functions have also been linked to difficulties in acquiring such abilities. Individuals with "weak mathematical performance of developmental origin" [29] have impairments in verbal and visual working memory [30–33], inhibition [33], and shifting [24]. Following the traditional model of working memory [34], some studies have focused on comparing whether individuals with dyscalculia have impaired verbal or visual working memory that leads to different results. For instance, Ashkenazi, Rosenberg-Lee, Metcalfe, Swigart, and Menon [35] reported that children with mathematical disabilities have preserved verbal working memory skills, but also have deficits in visuospatial working memory that can be observed at both the cognitive and functional neuroanatomical levels. Moreover, some authors have suggested that visual working memory is a predictor for math abilities, but not for reading skills [36]. More recent studies have begun to investigate the full domain of executive functions. For instance, difficulties in performing arithmetic operations have been associated with impaired working memory and inhibition: working memory serves as a mental workspace for the manipulation of operators, operands, and numerical facts, and inhibition could suppress irrelevant information [33]. Working memory has also been linked with fact retrieval, another major impairment in children with MLD, which determines how much information is rehearsed and could also influence how much of this information is consolidated as long-term memory representation [37].

However, there is a need for further study of the link between executive functions and mathematical talent. For instance, indirect evidence suggests that high performance in math is related to an ability to alternate efficiently between strategies. By comparing the brain activity patterns of an expert calculator and six non-expert adults performing multi-digit multiplications, Pesenti and colleagues [38] found that experts switched between brain areas involved in computing and those involved in fact retrieval. More recently, Swanson [39] compared the performance of children scoring 1.5 SD above the mean on two standardized mathematical tests and those with average scores on a series of tests including measures of short-term memory, working memory, and inhibition. In his study, mathematical talent was associated with a latent variable representing working memory, which included the digit/sentence, backward digit, listening/sentence, and updating tasks. In a review of current literature on the cognitive correlates of mathematical talent, Myers, Carey, and Szucs [40] concluded that mathematical talent is associated with spatial processing, working memory, efficient strategy switching, and inhibition. Overall, working memory has been the component of executive function consistently found to contribute to both ends of the distribution of mathematical abilities [18,40].

### The study

Are mathematical learning difficulties and mathematical talent related to the same underlying abilities? That is, are these two categories the lower and upper ends of the normal distribution of mathematical aptitude? Or are they discontinuous, qualitatively different cases? To study mathematical learning difficulties, researchers have traditionally taken the disability/ability approach, where difficulty is understood as the result of an impaired cognitive ability (e.g., number sense, working memory) or the malfunction of a specific brain region or regions [41–43]. Recent approaches have suggested that different levels of the same abilities whose deficits are responsible for learning disabilities may also explain the normal variation [30,44]. Here, we take a step further and suggest that a heightened degree of these same abilities could also be responsible for exceptional mathematical aptitude.

**Fig 1:**
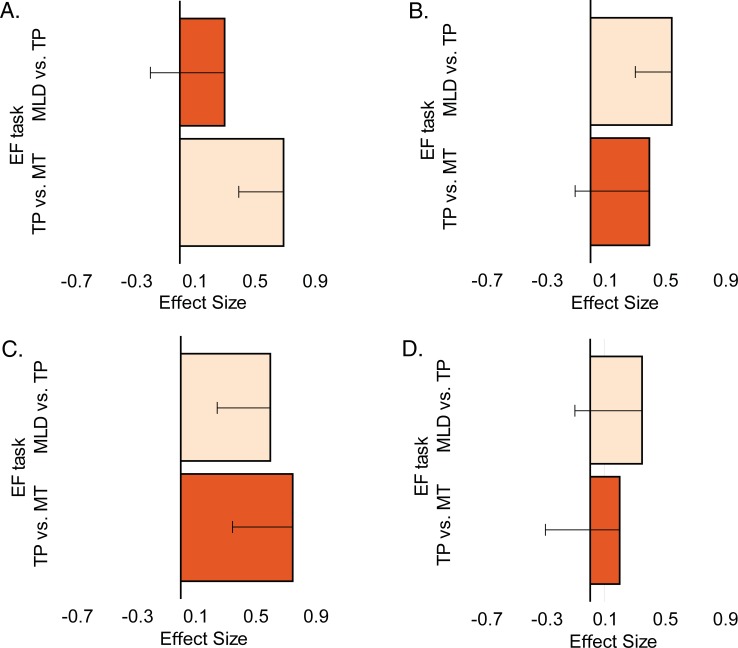
Patterns of hypothetical contributions of executive functions (EF) to the whole continuum of the mathematical abilities. (A) Contribution to Mathematical Talent (MT), (B) contribution to Mathematical Learning Difficulties (MLD), (C) contributions to both ends of math performance, and (D) no contributions.

To obtain empirical support for this idea, we evaluated three groups of adolescents with distinct mathematical achievement levels: a mathematical learning difficulties (MLD) group, a typical performance (TP) group, and a mathematical talent (MT) group. Participants in the three groups had similar reading speeds, verbal and non-verbal intelligence, and processing speeds. They were evaluated with a suite of executive function tasks (verbal and visuospatial working memory, inhibition, and shifting) and with verbal and visuospatial short-term memory tasks. Given the associations found between mathematical aptitude or learning difficulties and individual differences in verbal and visual working memory, we predicted that working memory would contribute to both ends of the distribution: that is, that adolescents in the MT group would outperform those in the TP group, who would in turn outperform those in the MLD group. Fig 1 shows the hypothetical patterns for the contributions of different executive functions to mathematical performance. Fig 1C provides a visual representation of our predicted pattern for the measures of working memory: effect sizes for both pairwise comparisons (MLD vs. TP and TP vs. MT) will be significant.

## Methods

### Participants

A total of 2,682 first year high school students aged 14 years to 16 years 11 months, from three public schools in Guadalajara, Mexico, were screened for mathematical abilities using the WRAT Math Computation subtest [12]. A subsample of 74 students and their parents gave written informed consent for an expanded assessment. This evaluation included the Vocabulary (verbal intelligence), Matrix Reasoning (non-verbal intelligence), and Arithmetic subtests from the Mexican version of the WISC-IV [45]; the Written Math and the Reading a Text Aloud subtests from the *Evaluación Neuropsicológica Infantil* (ENI) [Neuropsychological Assessment for Children] [46]; and a suite of executive function and short-term memory tasks. Forty-eight adolescents met criteria for one of three groups of interest that formed the final sample: MLD (n = 16, mean age = 16.38 [SD = 0.69], 56% girls), TP (n = 16, mean age = 16.28 [SD = 0.99], 56% girls), and MT (n = 16, mean age = 16.28 [SD = 0.92], 50% girls). The groups were similar in their mean age: *F*(2, 45) = 0.75, *p* = .92, η^2^ = .003.

For the MLD group, the criteria included: (a) a score at least 1.5 SD below the mean for Mexican first-year high school students [10] on the WRAT Math Computation subtest, and (b) a score below the 10th percentile on the ENI Written Math subtest. For the TP group, the criteria included (a) a score within the range of ± 1 SD of the Mexican standard mean on the WRAT Math Computation subtest, and (b) a score above the 10th percentile on the ENI Written Math subtest. Finally, for the MT group, the criteria included (a) a score ≥ 1.5 SD above the Mexican standard mean on the WRAT Math Computation subtest, and (b) a scaled score above the 90th percentile on the ENI Written Math subtest. Participants from all groups were required to have (c) a scaled score ≥ 7 on the WISC Vocabulary subtest, (d) a scaled score above the 10th percentile on the ENI Reading a Text Aloud subtest, and they were required (e) not to meet the diagnostic criteria for ADHD based on a parent questionnaire [47].

**Fig 2:**
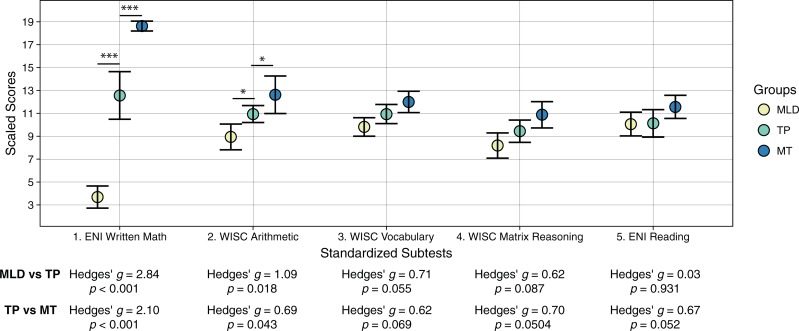
Mean scaled scores for each group of criterion subtests. Group means and 95% confidence intervals are shown. Below the X-axis, effect sizes (Hedges' *g*) are given for the difference between the pairwise comparisons across groups. MLD = Mathematical learning difficulties, TP = Typical performance, MT = Mathematical talent. * *p* < .05, *** *p* < .001.

The TP group was used as the comparison group: adolescents from MLD and MT groups had similar age, reading skills, and verbal and non-verbal intelligence as those in this group. Fig 2 summarizes performance of each group for the standardized subtests. To further describe their math abilities, Table 1 shows the mean correct responses of each group on the WRAT Math Computation subtest and the mean proportion of correct responses for each of the three factors of this subtest described in Abreu-Mendoza et al. [10]. Across these factors, the largest significant difference between the MLD and TP groups was in the Arithmetic factor, while the largest difference between the MT and the TP groups was in the Rational Numbers factor.

**Table 1:** Mean proportion (standard deviation) of correct responses on the WRAT math computation subtests by group, and effect size (Hedges' *g*) for the differences between groups.

	WRAT: Total Score	Factor 1: Fractions and Basic Algebra	Factor 2: Rational Numbers	Factor 3: Arithmetic
	M (SD)	M (SD)	M (SD)	M (SD)
MLD	14.19 (2.04)	0.12 (0.12)	0.03 (0.07)	0.17 (0.15)
TP	26.37 (2.45)	0.56 (0.22)	0.10 (0.13)	0.76 (0.17)
MT	35.44 (1.42)	0.94 (0.06)	0.70 (0.17)	0.92 (0.073)
	Hedges' *g* [97.5%]	Hedges' *g* [97.5%]	Hedges' *g* [97.5%]	Hedges' *g* [97.5%]
MLD vs TP	**5.27 [3.55, 6.68]**	**2.43 [1.33, 3.47]**	0.67 [-0.12, 1.39]	**3.579 [1.88, 5.24]**
TP vs MT	**4.42 [3.00, 6.041]**	**2.33 [1.45, 3.46]**	**3.72 [2.41, 5.21]**	**1.19 [0.42, 1.92]**

Note: MLD = Mathematical learning difficulties, TP = Typical performance, MT = Mathematical talent.

The University of Guadalajara ethics committee approved this research study (approval number ET112015-205), which included verification that the consent form, procedures, and methods were in compliance with the Helsinki principles.

### Experimental tasks

To cover the whole range of executive functions proposed by Miyake and colleagues [14,48] and both components of working memory (updating and capacity) in its two modalities (verbal and visual), we used established tasks as outcome measures of these functions.

#### Digit span

Verbal short-term and working memory spans were assessed with the Digit Span subtest from the Mexican version of the WISC-IV [45]. The dependent variables for verbal STM and working memory were the longest sequence of each condition, forward and backward, respectively.

#### Simple reaction time task

To evaluate speed processing, adolescents were asked to perform a simple reaction time (RT) task, in which they had to press a spacebar as soon as they saw the letter "A" appear on a screen. The letter was shown with a random interstimulus interval (ISI) of 500–2000 milliseconds over 30 trials. The dependent variable was the mean reaction time of the 30 trials.

#### Corsi block-tapping task

The span of visual short-term and working memory were measured with the computerized version of the Corsi block-tapping task [49] from the Psychology Experiment Building Language (PEBL) test battery [50]. The dependent variables for visual STM and working memory were the longest sequence of each condition, forward and backward, respectively.

#### Letter n-back task

The updating component of verbal working memory was evaluated with two memory load (1- and 2-back) conditions of the letter n-back task [51]. The 1-back condition consisted of 50 trials and the 2-back of 90 trials. Participants had to press the space bar as soon as they saw the same letter as the one just shown (1-back) or the one preceding it (2-back). The stimulus duration was 700 milliseconds and the ISI was 500 milliseconds in both conditions, and the proportion of targets was 0.3. The dependent variable was the Matthews correlation coefficient [52]. This accuracy measure uses all four response types—true positives (TP), true negatives (TN), false positives (FP), and false negatives (FN)—and thus takes into account trade-offs between these responses:

$$Mathews\;coefficient=\frac{TP*TN-FP*FN}{\sqrt{\left(TP+FN)(TP+FP)(TN+FP)(TN+FN\right)}}$$

#### Visual n-back task

The updating component of visual working memory was evaluated with a task similar to the letter n-back task, but instead of seeing a sequence of letters, participants saw a sequence of asterisks on a screen that could appear in one of four positions (upper right, upper left, lower right, and lower left). Participants had to press the space bar as soon as they saw the asterisk in the same position as the one just shown (1-back) or the one preceding it (2-back). In both the 1-back and 2-back conditions the stimulus duration was 700 milliseconds, the ISI was 500 milliseconds, and the proportion of targets was 0.3. The dependent variable was the Matthews correlation coefficient.

#### Go/no-go task

To measure adolescents' inhibition of preponderant responses, we used a go/no-go task, which consisted of two parts. In the go part, participants had to press a spacebar as soon as they saw a letter on a screen; in the no-go part, they had to press the spacebar when they saw any letter except the letter "J." The first part consisted of 50 trials and the second 150 trials. The proportion of no-go trials was 0.3. In both parts, the stimulus duration was 700 milliseconds and the ISI was 500 milliseconds. The dependent variable was the Matthews correlation coefficient.

#### Local-global task

Adolescents' ability to shift between task instructions was assessed with a local-global task consisting of three conditions. In the first, participants saw a sequence of blue Navon figures ("global" figures, for example squares, whose lines are composed of smaller, "local" figures, for example triangles). They were asked to indicate the number of sides of the global figure. In the second condition, participants saw red Navon figures and were asked to indicate the number of sides of the local figure. In the third condition, the shifting condition, participants saw both blue and red Navon figures and were asked to indicate the number of sides of the global or local figure, according to the rules learned in the two previous conditions (blue = number of sides of the global figure, red = number of sides of the local figure). The number of trials per condition was 31, 31, and 105, respectively, and the dependent variable was the proportion of correct responses in the shifting condition.

### Procedure

Criterion subtests were administered in the following fixed order: the Vocabulary, Matrix Reasoning, and Arithmetic subtests of the WISC-IV, followed by the Reading a Text Aloud and Written Math subtests of the ENI; they were administered either in participants' schools or in our lab. Those taking the criterion subtests at their schools performed the experimental tasks in a second session in our lab; those taking the tests in the lab were given a five-minute break before the experimental tasks. The tasks were presented in the following order: digit span, RT, Corsi-block tapping tasks; the letter n-back, go/no-go, visual n-back, and local/global tasks were then presented in a counterbalanced order without presenting the n-back tasks consecutively. The RT task was programmed and presented using E-prime 2.0, and the last four tasks were programmed using Psychtoolbox running under MATLAB. All computerized tasks were presented on a 23-inch LED monitor and participants were seated approximately 65 cm from the monitor. The administration of the criterion subtests lasted approximately 40 minutes, and the experimental tasks lasted about an hour. At the end of the session, families received a travel reimbursement of approximately $10 USD.

### Statistical analyses

After confirming the absence of multivariate outliers using the Mahalanobis distance and verifying homogeneity with Levene's test (Levene's *p* > .001), we performed statistical analyses for each of the evaluated cognitive constructs: reaction time, short-term memory, and executive functions. For the simple reaction time task, we performed a one-way ANOVA with Group (MLD, TP, and MT) as between-subjects factor.

To compare performance across the three groups on the two short-term memory and six executive function tasks, we performed two independent, one-way MANOVAs with Group (MLD, TP, and MT) as between-subjects factor. In a first step, we tested the MANOVA assumption of correlation between the dependent variables by performing a series of Pearson correlations with the scores of each of the two types of tasks. The second step was to introduce these scores as the dependent variables of the one-way MANOVAs with Group as the between-subjects factor. In the third step, we conducted a series of follow-up ANOVAs to determine the subtest scores in which the main effect was present. Finally, for ANOVAs that were significant after correcting for multiple comparisons using Holm correction, we performed least significant difference (LSD) post-hoc tests between the MLD and TD groups, and between the MT and TD groups.

Mindful of recent criticism of the use of *p*-values [53], we also calculated standardized effect sizes and bootstrapped confidence intervals (CIs). Confidence intervals not only indicate whether a result is statistically significant, they also communicate the precision of the effect size and can be considered significant if they do not include zero. Parametric methods for calculating CIs make several assumptions about the shape of the data distribution. To avoid these assumptions, bootstrap methods approximate the distribution from the data itself by repeatedly drawing samples, with replacement, from the original data sample. Here we calculated bootstrapped, bias-corrected and accelerated (BCa) confidence intervals using the bootES package [54] of the statistical programming language *R*, utilizing 100,000 permutations with replacement.

We used Hedges' *g* as a measure of effect size, as it is more appropriate for the estimation of unbiased effect sizes in studies with small sample sizes (*n* < 20) than Cohen's *d* [53,55]. As Holm correction cannot be used to determine adjusted confidence intervals, we used a Bonferroni correction, which resulted in 97.5% and 99.375% CIs for the short-term memory and executive function tasks, respectively. Depending on the task, effect sizes (Hedges' *g*) were interpreted as being significant if the 97.5% or the 99.375% bootstrap CIs excluded zero. Using similar guidelines as those for Cohen’s *d* [56], Hedges’ *g* values of 0.2, 0.5, and 0.8 can be considered small, medium, and large effects, respectively.

A post hoc sensitivity power analysis showed that our sample had sufficient power (β = .80) at a significance level of ɑ = .05 to detect medium to large effect sizes (f ≥ .463) in a one-way ANOVA, and to detect large effect sizes (Cohen's *d* ≥ 1.02) in an independent sample *t*-test. Power analysis was done with G-Power [57]; all statistical analyses were performed using R [58].

## Results

**Fig 3:**
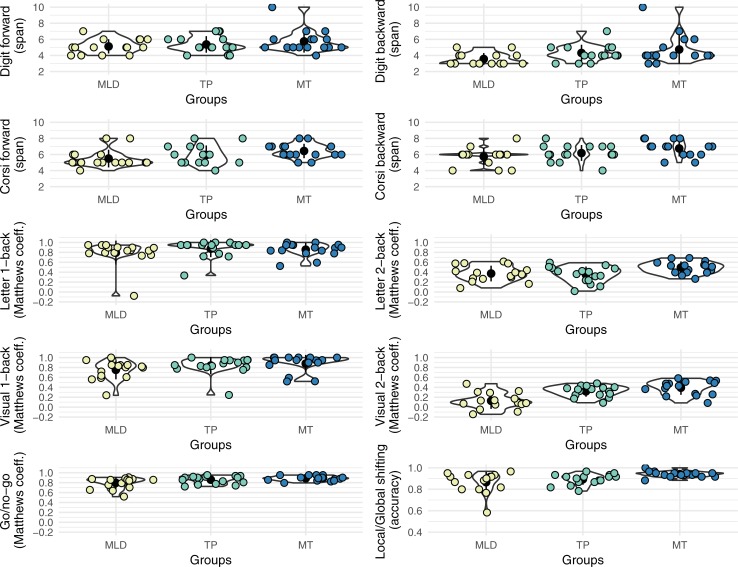
Violin plots for the two short-term memory and six executive function tasks. Each dot represents one participant. MLD = Mathematical learning difficulties, TP = Typical performance, MT = Mathematical talent.

**Fig 4:**
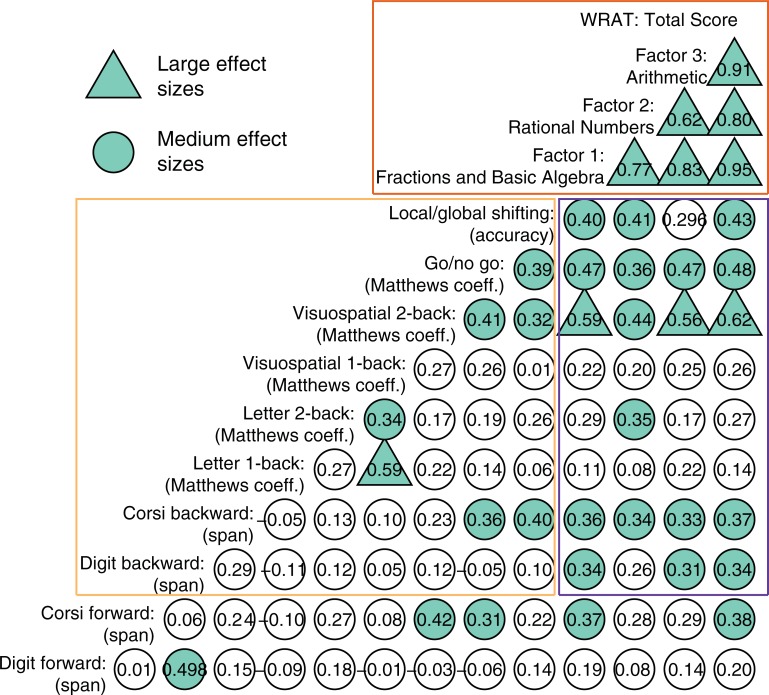
Pearson correlation coefficients between the executive function tasks (yellow rectangle), between the factors of the math computation subtest (orange rectangle), and between the executive function tasks and math factors (blue rectangle). Correlations with values > .3 have *p* < .05; correlations with values > .4 have *p* < .01; correlation with values > .5 have *p* < .001.

To visualize the distributions of the scores of the two short-term memory and the six executive function tasks, Fig 3 shows violin plots and data points for all participants across these tasks. Descriptive data on all measures for the whole sample and for each group are presented in Tables A to D in S1 Appendix. Visual inspection of the plots suggests a linear trend across groups: adolescents in the MT group showed higher scores than those of the TP group, which in turn had higher scores than those of the MLD group. The correlation coefficients in Fig 4 confirm these impressions. There were small to large correlation coefficients between the number of correct responses on the Math Computation subtest and the scores in the EF tasks. However, these correlations might be driven by the differences in Vocabulary and Matrix reasoning scores between the MLD and TP groups.

Fig 4 also shows the correlations between the executive function tasks, between the factors from Math Computation subtest, and between the executive function tasks and the math factors; overall, there were small to medium correlations between the executive function tasks and between these tasks and factors of the WRAT Math Computation subtest as well.

### Simple reaction time task

The ANOVA showed no significant effect of Group on reaction times: *F*(2, 45) = 1.46, *p* = 0.24, η^2^ = 0.06.

### Short-term memory tasks

Table 2 shows the descriptive statistics for these tasks. The MANOVA did not show a main effect of group: Pillai's Trace = 0.17, *F*(4, 90) = 2.12, *p* = .08, η^2^ = .10.

**Table 2:** Mean (standard deviation) for the simple RT task and the two short-term memory tasks.

	MLD	TP	MT			
	M (SD)	M (SD)	M (SD)	F(2,45)	*p*-value	ηp^2^
Reaction Time (ms)	303.79 (60.40)	275.73 (39.33)	282.19 (43.40)	1.464	0.24	0.06
Digit forward (span)	5.12 (0.88)	5.37 (1.02)	5.75 (1.39)	1.261	0.29	0.05
Corsi forward (span)	5.50 (1.09)	5.94 (1.18)	6.44 (0.89)	3.114	0.05	0.12

Note: MLD = Mathematical learning difficulties, TP = Typical performance, MT = Mathematical talent. Table shows uncorrected *p*-values.

### Executive function tasks

The MANOVA yielded a main effect of Group: Pillai's Trace = 0.74, *F*(16, 78) = 2.87, *p* = .001, η_p_^2^ = 0.45. Univariate ANOVAs were used to examine individual dependent variable contributions to main effects. After controlling for multiple comparisons, only the performance of the go/no-go task, *F*(2, 45) = 5.34, *p =* .0499, η_p_^2^ = 0.19, the visual 2-back condition, *F*(2, 45) = 15.55, *p* < .001, η_p_^2^ = 0.41, and the local global task, *F*(2, 45) = 5.61, *p* = .047, η_p_^2^ = 0.20, showed a main effect of group. Detailed statistics for each of the ANOVAs are shown in Table 3.

**Table 3:** Mean Matthews correlation coefficient and mean accuracy (%) with 97.5% confidence intervals for executive function tasks.

	MLD	TP	MT			
	M (SD)	M (SD)	M (SD)	*F*(2,45)	*p*-value	ηp^2^
Digit backward (span)	3.56 (0.73)	4.31 (1.01)	4.75 (1.77)	3.69	0.033	0.14
Corsi backward (span)	5.75 (1.06)	6.2 (0.98)	6.75 (1.00)	3.90	0.028	0.15
Letter 1-back (Matthews coeff.)	0.79 (0.24)	0.87 (0.17)	0.85 (0.13)	0.80	0.456	0.03
Letter 2-back (Matthews coeff.)	0.37 (0.16)	0.35 (0.16)	0.49 (0.12)	4.39	0.018	0.16
Visual 1-back (Matthews coeff.)	0.75 (0.19)	0.84 (0.17)	0.88 (0.17)	2.08	0.136	0.08
Visual 2-back (Matthews coeff.)	0.13 (0.16)	0.32 (0.11)	0.39 (0.14)	15.55***	0.000	0.41
Go/no-go (Matthews coeff.)	0.79 (0.11)	0.86 (0.08)	0.89 (0.05)	5.34*	0.008	0.19
Local/Global shifting (acc)	0.87 (0.10)	0.89 (0.05)	0.94 (0.03)	5.61*	0.007	0.20

Note: MLD = Mathematical learning difficulties, TP = Typical performance, MT = Mathematical talent. Table shows uncorrected *p*-values. Asterisks depict statistically significance after correcting for multiple comparisons.

* *p* < .05

*** *p* < .001

In the go/no-go task, least significant difference (LSD) post hoc analyses for the planned pairwise group comparisons showed that the TP group (M = 0.86, SD = 0.07) outperformed the MLD group (M = 0.79, SD = 0.11, *p* = 0.03). In the visual 2-back task, the TP group (M = 0.32, SD = 0.11) also outperformed the MLD group (M = 0.13, SD = 0.16, *p* < .001). Finally, in the local/global task, the MT group (M = 0.89, SD = 0.05) outperformed the TP group (M = 0.94, SD = 0.03, *p* = .04).

### Effect sizes and bootstrapped confidence intervals

**Fig 5:**
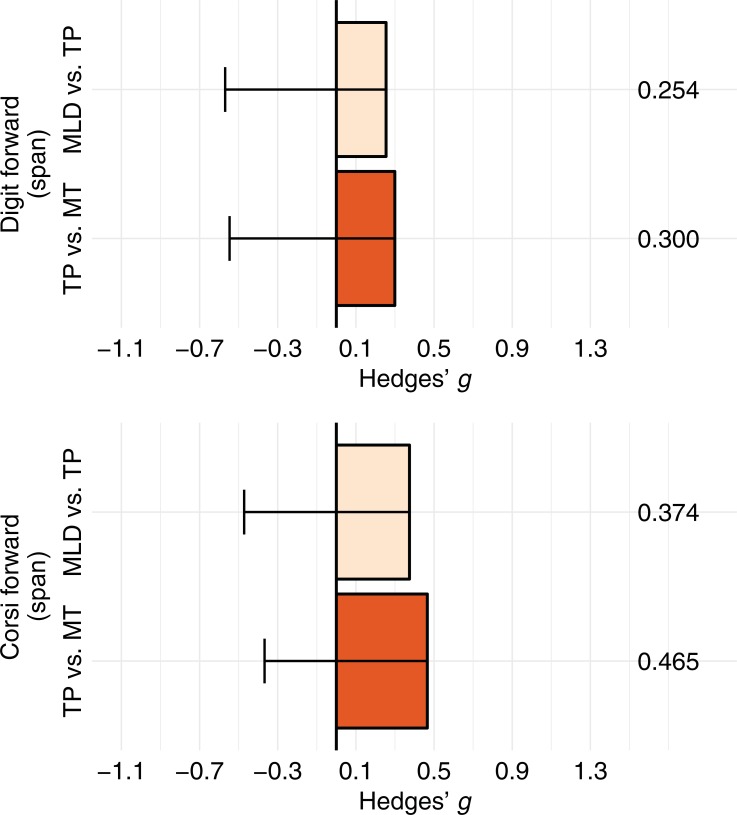
Group differences (expressed in Hedges' *g*) for the two short-term memory subtests: Digit subtest (forward condition) and corsi block tapping test (forward condition). Error bars represent the adjusted 97.5% bootstrapped CI (only the lower level is shown). Effect sizes are significant if error bars do not include zero. MLD = Mathematical learning difficulties, TP = Typical performance, MT = Mathematical talent.

**Fig 6:**
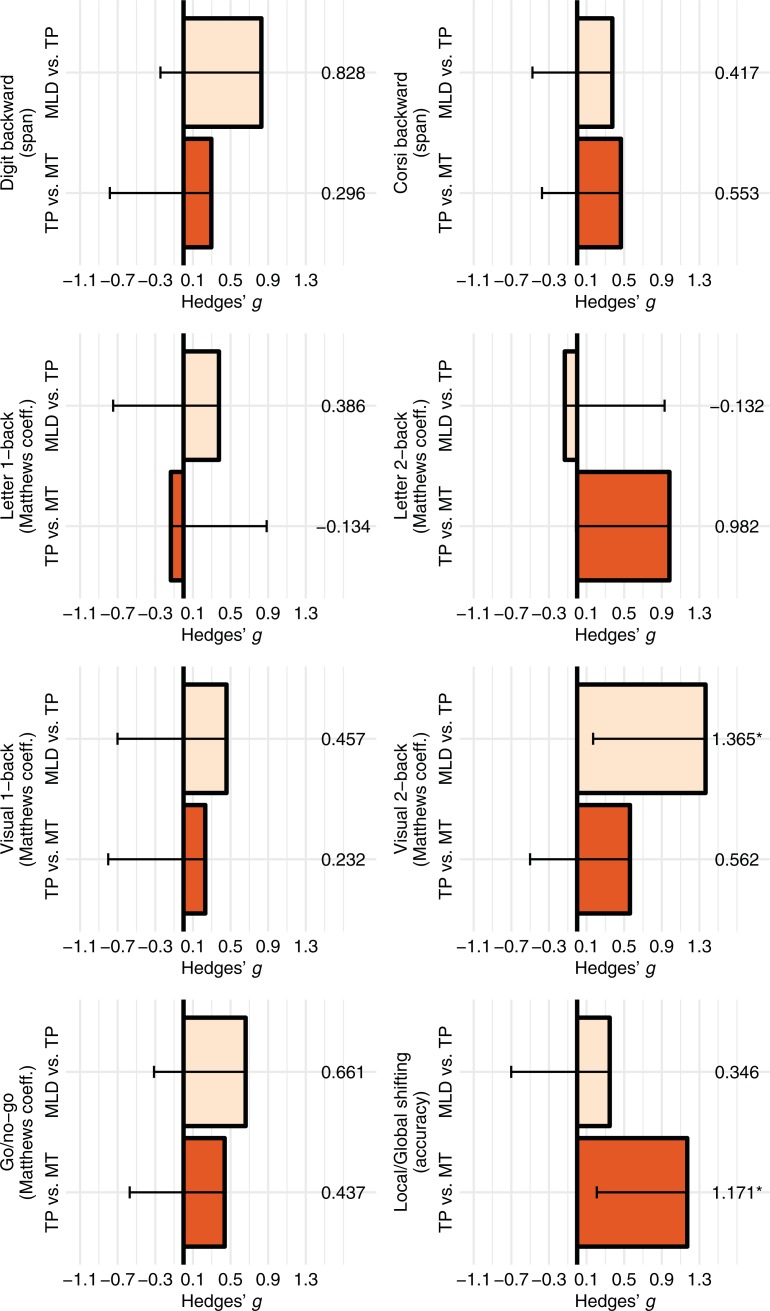
Group differences (expressed in Hedges' *g*) for the six executive functions tasks. Error bars represent the adjusted the 99.375% bootstrapped CI (only the lower level is shown). Effect sizes are significant if error bars do not include zero. MLD = Mathematical learning difficulties, TP = Typical performance, MT = Mathematical talent. * *p* < .05.

Figs 5 and 6 summarize the effect sizes (Hedges' *g*) and their bootstrapped confidence intervals of the group differences (TP minus MLD and MT minus TP) in each of the short-term memory and executive function tasks. Consistent with the parametric tests, confidence intervals did not include zero for the effect sizes for the differences between the TP and MT groups in the local/global task or the differences between the MLD and TP groups in the visual 2-back task. According to Cohen's criteria [56], these effect sizes can be considered large. These effect sizes remained significant when outliers (> 2 *SD* from the groups’ mean) were removed from the analyses: visual 2-back task, MLD (n = 15) vs. TP (n = 15) Hedges' *g* = 1.22 [0.16, 2.18]; local/global task, TP (n = 15) vs. MT (n = 15) Hedges’ *g* = 1.17 [0.23, 2.05]. Furthermore, regression line analyses, using the Math Computation scores as a continuous variable, showed a similar pattern: the slope for the visual 2-back task scores was significant only for the Math Computation scores within the range of the MLD and TP groups (see Fig A, upper panel, in S1 Appendix), while the slope for the local/global task scores was significant only for the Math Computation scores within the range of the TP and MT groups (see Fig A, lower panel, in S1 Appendix).

It is noteworthy that even though the *p-*value for the differences between the TP and MT groups in the local global task was relatively large (*p* = .04), the effect size for this difference can be considered large (Hedges' *g* = 1.17). As *p*-values are more vulnerable to sample size than effect sizes, we believe the group differences are best interpreted based on the latter, as well as on confidence intervals. Finally, even though the effect size for the outperformance of the TP group relative to the MLD group in the go/no-go task was medium (Hedges' *g* = 0.661 [-0.32, 1.54]), its confidence interval included zero, suggesting that this result has low precision.

## Discussion

This study aimed to ascertain whether the same executive functions contribute to individual differences in math ability, ranging from impairment (MLD group) to talent (MT group). Researchers have suggested that individuals with learning disabilities can be seen either as qualitatively different (having a specific impairment in a cognitive ability) or as quantitatively different (the ability associated with normal variation is associated with a learning difficulty). Contrary to our predictions, our results suggest that different executive functions contribute to mathematical learning difficulties and mathematical talent. Our group of adolescents with MLD had a lower level of updating visuospatial abilities than the control (TP) group. In contrast, those with mathematical talent were distinguished from the typical performance group for their greater ability to shift between instructions. Consistent with some theoretical proposals that distinguish the contribution of executive functions to behavioral performance from that of intelligence and processing speed, e.g., [59], these differences are not the result of variations in other cognitive abilities—verbal and non-verbal intelligence, reading speed, or processing speed. There were no differences across groups in these domains, and their effect sizes were smaller than those reported for the differences between groups in the executive function tasks.

Another important finding of our study is that adolescents’ differences in mathematical performance are not reflected in all types of mathematical abilities. Those in the MLD group were mostly distinguished from their peers by difficulty in performing arithmetic problems with whole numbers and decimals (Factor 3: Arithmetic), while those in the MT group stood out for their ability to solve arithmetic problems with fractions (Factor 2: Rational Numbers). Surprisingly, there were no differences between the MLD and typical performance groups in this last factor, suggesting that adolescents at this academic stage may be misclassified if evaluated only on the basis of their ability to perform arithmetic with fractions.

Studies have shown that the strength of the relationship between executive function tasks varies across development. While there is a strong association between its different components in early developmental stages, these functions are more differentiated during adolescence, and their correlation coefficients are not as high as in childhood [18]. Similarly, the correlation coefficients observed in our data show only small to moderate associations between the different measures (r = 0.32–0.40), suggesting that working memory, inhibition, and switching are well differentiated at the ages of 16 and 17, and that it is feasible to assess the contribution of each domain in mathematical achievement.

Consistent with previous studies [31,60], our results suggest that there is an interplay between different cognitive skills and executive functions and the level achieved in solving specific types of mathematical problems. Research has shown that working memory and inhibition, but not switching, are associated with individual differences in solving problems with whole numbers [26]. Working memory, especially visuospatial memory, may play a crucial role in maintaining information when performing multi-digit arithmetic problems: in such problems, not only the intrinsic value of the digit, but also its position convey information of its magnitude. Other authors have also suggested that maintaining information is a crucial step in forming long-term representations of arithmetical facts [37]. Therefore, the impaired visuospatial working memory that characterized our MLD group, an impairment that has been noted in several studies, see [32] for a review, may explain why the largest difference relative to their typically performing peers was in their ability to perform multi-digit arithmetic.

The links between the superior performance of the MT group on the local/global task and their greater ability to solve problems involving fractions with unlike denominators are less clear. How is the ability to alternate between different instructions or mindsets related to a greater ability to manipulate fractions? One possible link is the representational flexibility of fractions. Rational numbers can be represented in infinite ways, some more convenient than others, while performing a specific operation. Consider the sum of the fractions 5/15 + 4/12. For students who have learned that the first step in solving these problems is to obtain a common denominator, typically the product of the two denominators, this problem may seem overwhelming. However, students with more flexible strategies may realize that this operation is just another form of the simple sum of 1/3 + 1/3. Indirect evidence for this explanation comes from a recent study [61] showing that college students with high mathematical proficiency used a large variety of strategies to compare the magnitude of two fractions. Most importantly, asked to choose the best strategy from among three possibilities, students with high mathematical proficiency changed their original incorrect response to the correct one more often than those with less mathematical knowledge.

Another link between shifting and fractional arithmetic may be related to the partial overlap between the procedures for solving problems involving whole numbers and fractions. As whole number procedures allow solutions only in specific cases (e.g., addition and subtraction of fractions with like denominators), students have to learn when to switch from these procedures to those that apply to fraction arithmetic. Applying whole number procedures is a frequent error in fraction arithmetic [62]. This requirement to switch strategies may explain why adolescents with greater mathematical abilities also had greater shifting abilities than those with typical performance.

All of these links between executive functions and mathematical abilities refer to the online contribution of these functions in solving mathematical problems. However, executive functions could be contributing to mathematical learning in more extensive ways, by supporting the conceptual changes that occur in children’s and adolescents’ mathematical knowledge. Mathematical knowledge is learned not only by enriching prior knowledge, but also through qualitative changes [63,64]; that is, individuals not only become more proficient in certain abilities, but also need to develop new concepts. One of these changes relates to one of the most fundamental concepts in mathematics: the concept of number. According to some authors, the concept of natural number builds on the core systems for number [65,66], while that for rational numbers builds on some of the intuitions that children have about natural numbers [67]. Importantly, the early concepts continue to exist throughout development and are faster and easier to activate than recently acquired, unstable concepts [68]. To successfully go through these changes, individuals will thus have to *inhibit* previous concepts and be able to *switch* to the newer set of rules.

Only a small number of studies have looked at the relationship between executive functions and conceptual change in other domains, especially during childhood. For instance, measures of inhibition have been shown to predict false-belief understanding, a component of theory of mind [69], and a composite of executive functions tasks have been associated with children’s understanding of vitalist biology (understanding of life, death, and body functions) [70]. Using assessments that tap into conceptual understandings [71,72], future studies could explore more comprehensively the role of executive functions in mathematical knowledge, not only in childhood but in adolescence as well.

These conclusions should be considered in light of the limitations of this study, one of which is its low statistical power to detect medium and small effect sizes. According to the power analysis, our statistical power to detect medium effect sizes (Cohen’s *d* = 0.5) was 0.19, while the power to detect small effect sizes (Cohen’s *d* = 0.2) was 0.05. As highlighted by Szucs and Ioannidis [73], underpowered studies not only lead to false negatives (i.e., failing to detect an existing effect), but also to exaggeration of measured effect sizes. For these reasons, it is important to keep confidence intervals in mind when looking at “significant” results, as they provide less biased information than *p*-values [53]. Among the multiple concerns with assessing executive functions is the so-called task impurity problem: it is difficult to isolate an index of a single component, given that tasks also trigger additional executive and non-executive processes [74]. To minimize this problem, researchers have used a latent variable approach, extracting the common variance among tasks that measure the same function. This approach also prevents the specific requirements of the tasks from driving the association with the predicted variable. However, the disadvantage of this approach is that it requires large samples (n > 100).

In our study, it is uncertain whether the association between executive function tasks and mathematical scores is due to their underlying executive components or to the complexity or specificity of their demands. We addressed this problem in part by measuring the different components (span and updating) and modalities (verbal and visuospatial) of working memory, but could not do so for inhibition and shifting. In order to address the task impurity problem in these latter executive components, we isolated a specific executive function using simple tasks, such as the go/no-go task, but some individuals in each of the three groups reached a performance ceiling. This limitation, along with the inability to detect medium effect sizes, could have contributed to the lack of differences between groups in our inhibition measure (go/no-go task). Previous studies have shown that inhibition scores using larger sample sizes (N = 127) [39] or more complex measures (e.g., Stroop task) [33] can distinguish individuals with typical performance from those at either end of the math ability continuum. However, complex measures have higher levels of task impurity, making it difficult to isolate EF components. The Stroop task, for example, has loaded on factors involving set-shifting [75].

## Conclusions

This study aimed to investigate whether the same executive functions contribute to the continuum of mathematical abilities in adolescence or whether they make specific contributions to mathematical learning difficulties and mathematical talent. Against our expectations, there were executive functions that had a larger contribution at one end of the continuum than the other. There were differences in the updating component of visual working memory between the group of adolescents with MLD and those with typical performance, but not between those with typical performance and mathematical talent. However, the ability to shift between instructions contributes to the distinction between those adolescents with mathematical talent and those with typical performance. Our results also suggest that there is an interplay between these ability levels, shown in the different components of executive function and specific types of mathematical problems.

## Supporting information

S1 Appendix This appendix contains Tables A-D and Fig A. (DOCX)
