# Supplementary material for: The contributions of executive functions to mathematical learning difficulties and mathematical talent during adolescence
Source: PLoS One. 2018 Dec 13;13(12):e0209267. doi: 10.1371/journal.pone.0209267 (PMC6292664; doi:10.1371/journal.pone.0209267)
Supplement: S1 Appendix — (DOCX) [file pone.0209267.s001.docx]

**PLoS One Supporting Information Appendix S1**

Article title: The Contributions of Executive Functions to Mathematical Learning Difficulties and Mathematical Talent During Adolescence

Authors: Abreu-Mendoza, R. A., Chamorro, Y., Garcia-Barrera, M. A., & Matute, E.

The following Supporting Information is available for this article:

**S1 Table A. Descriptive data of the whole sample (N = 48)**

**S1 Table B. Descriptive data of the MLD group (n = 16)**

**S1 Table C. Descriptive data of the TP group (n = 16)**

**S1 Table D. Descriptive data of the MT group (n = 16)**

**Fig A. Scatterplots for the correlation between the WRAT Math Computation subtest and accuracy in the visual 2-back task (upper panel) and the local/global task (lower panel).** Red lines show the regression lines considering only the Math Computation scores of the MLD and TP groups, while black lines consider only those of the TP and MT groups. **p < .01

**S1 Table A. Descriptive data of the whole sample (N = 48)**

| Measures | M | SD | Skewness | Kurtosis |
| --- | --- | --- | --- | --- |
| Criterion measures |  |  |  |  |
| ENI Written Math | 11.63 | 6.67 | -1.67 | -0.21 |
| WISC Arithmetic | 10.83 | 2.72 | -0.14 | 0.18 |
| ENI Reading | 10.58 | 2.11 | -0.48 | 0.03 |
| WISC Vocabulary | 10.92 | 1.82 | -0.60 | 0.00 |
| WISC Matrix Reasoning | 9.50 | 2.27 | -0.68 | 0.31 |
| Reaction Time | 287.24 | 49.05 | 0.83 | 1.29 |
| Short term memory |  |  |  |  |
| Digit forward (span) | 5.42 | 1.13 | **4.50** | 1.52 |
| Corsi forward (span) | 5.96 | 1.11 | -0.68 | 0.48 |
| Executive Functions |  |  |  |  |
| Digit backward (span) | 4.21 | 1.32 | **7.15** | 2.20 |
| Corsi backward (span) | 6.23 | 1.08 | -0.08 | -0.27 |
| Go/no-go (Matthews coeff.) | 0.85 | 0.09 | 2.96 | -1.53 |
| Visual 1-back (Matthews coeff.) | 0.82 | 0.18 | 2.54 | -1.59 |
| Visual 2-back (Matthews coeff.) | 0.28 | 0.18 | -0.53 | -0.41 |
| Letter 1-back (Matthews coeff.) | 0.84 | 0.19 | **12.47** | -3.11 |
| Letter 2-back (Matthews coeff.) | 0.41 | 0.16 | -0.32 | -0.39 |
| Local/Global shifting (accuracy) | 0.90 | 0.07 | **7.83** | -2.29 |

**Note.** Values of skewness and kurtosis outside the appropriate range (< 3, <10, respectively; Brocki & Tillman, 2014; Kline, 2005) are marked in bold.

**Table B. Descriptive data of the MLD group (n = 16)**

| Measures | M | SD | Skewness | Kurtosis |
| --- | --- | --- | --- | --- |
| Criterion measures |  |  |  |  |
| ENI Written Math | 3.69 | 1.82 | 0.56 | 1.22 |
| WISC Arithmetic | 8.94 | 2.11 | -0.58 | 0.29 |
| ENI Reading | 10.06 | 1.95 | 1.51 | 0.89 |
| WISC Vocabulary | 9.81 | 1.52 | 0.22 | 0.36 |
| WISC Matrix Reasoning | 8.19 | 2.07 | 0.82 | 1.15 |
| Reaction Time | 303.79 | 60.40 | -0.50 | 0.95 |
| Short term memory |  |  |  |  |
| Digit forward (span) | 5.13 | 0.89 | -0.28 | 0.39 |
| Corsi forward (span) | 5.50 | 1.10 | 2.10 | 1.57 |
| Executive Functions |  |  |  |  |
| Digit backward (span) | 3.56 | 0.73 | -0.28 | 0.94 |
| Corsi backward (span) | 5.75 | 1.07 | 0.68 | -0.19 |
| Go/no-go (Matthews coeff.) | 0.79 | 0.11 | 1.06 | -1.34 |
| Visual 1-back (Matthews coeff.) | 0.75 | 0.19 | 2.30 | -1.31 |
| Visual 2-back (Matthews coeff.) | 0.13 | 0.16 | 0.18 | 0.42 |
| Letter 1-back (Matthews coeff.) | 0.79 | 0.24 | **12.99** | -3.46 |
| Letter 2-back (Matthews coeff.) | 0.37 | 0.16 | -0.73 | 0.00 |
| Local/Global shifting (accuracy) | 0.87 | 0.10 | **4.25** | -1.86 |

**Note.** Values of skewness and kurtosis outside the appropriate range (< 3, <10, respectively; Brocki & Tillman, 2014; Kline, 2005) are marked in bold.

**Table C. Descriptive data of the TP group (n = 16)**

| Measures | M | SD | Skewness | Kurtosis |
| --- | --- | --- | --- | --- |
| Criterion measures |  |  |  |  |
| ENI Written Math | 12.56 | 3.90 | -1.88 | -0.14 |
| WISC Arithmetic | 10.94 | 1.39 | 0.09 | 0.64 |
| ENI Reading | 10.13 | 2.25 | -0.61 | 0.10 |
| WISC Vocabulary | 10.94 | 1.57 | -0.21 | -0.24 |
| WISC Matrix Reasoning | 9.44 | 1.83 | -1.41 | -0.15 |
| Reaction Time | 275.73 | 39.33 | 0.36 | 1.24 |
| Short term memory |  |  |  |  |
| Digit forward (span) | 5.38 | 1.03 | -0.80 | 0.39 |
| Corsi forward (span) | 5.94 | 1.18 | -0.71 | 0.41 |
| Executive Functions |  |  |  |  |
| Digit backward (span) | 4.31 | 1.01 | 2.15 | 1.02 |
| Corsi backward (span) | 6.19 | 0.98 | 0.58 | -0.42 |
| Go/no-go (Matthews coeff.) | 0.86 | 0.08 | -1.16 | -0.58 |
| Visual 1-back (Matthews coeff.) | 0.84 | 0.17 | **9.65** | -2.83 |
| Visual 2-back (Matthews coeff.) | 0.32 | 0.11 | -0.27 | -0.59 |
| Letter 1-back (Matthews coeff.) | 0.87 | 0.17 | **6.52** | -2.41 |
| Letter 2-back (Matthews coeff.) | 0.35 | 0.16 | -0.44 | -0.55 |
| Local/Global shifting (accuracy) | 0.89 | 0.05 | 0.14 | -0.75 |

**Note.** Values of skewness and kurtosis outside the appropriate range (< 3, <10, respectively; Brocki & Tillman, 2014; Kline, 2005) are marked in bold.

**Table D. Descriptive data of the MT group (n = 16)**

| Measures | M | SD | Skewness | Kurtosis |
| --- | --- | --- | --- | --- |
| Criterion measures |  |  |  |  |
| ENI Written Math | 18.63 | 0.81 | 1.29 | -1.77 |
| WISC Arithmetic | 12.63 | 3.07 | 0.51 | -0.71 |
| ENI Reading | 11.56 | 1.90 | 1.27 | -0.75 |
| WISC Vocabulary | 12.00 | 1.75 | 0.47 | -0.60 |
| WISC Matrix Reasoning | 10.88 | 2.16 | -0.29 | 0.09 |
| Reaction Time | 282.19 | 43.40 | 2.49 | 1.50 |
| Short term memory |  |  |  |  |
| Digit forward (span) | 5.75 | 1.39 | **5.44** | 2.04 |
| Corsi forward (span) | 6.44 | 0.89 | -0.37 | 0.21 |
| Executive Functions |  |  |  |  |
| Digit backward (span) | 4.75 | 1.77 | **4.53** | 2.00 |
| Corsi backward (span) | 6.75 | 1.00 | -0.74 | -0.34 |
| Go/no-go (Matthews coeff.) | 0.89 | 0.05 | -0.76 | -0.17 |
| Visual 1-back (Matthews coeff.) | 0.88 | 0.17 | 0.94 | -1.55 |
| Visual 2-back (Matthews coeff.) | 0.39 | 0.14 | -0.37 | -0.68 |
| Letter 1-back (Matthews coeff.) | 0.85 | 0.13 | 1.65 | -1.38 |
| Letter 2-back (Matthews coeff.) | 0.49 | 0.12 | -0.59 | -0.10 |
| Local/Global shifting (accuracy) | 0.94 | 0.03 | 1.37 | 0.07 |

**Note.** Values of skewness and kurtosis outside the appropriate range (< 3, <10, respectively; Brocki & Tillman, 2014; Kline, 2005) are marked in bold.

**Supplementary Figures**


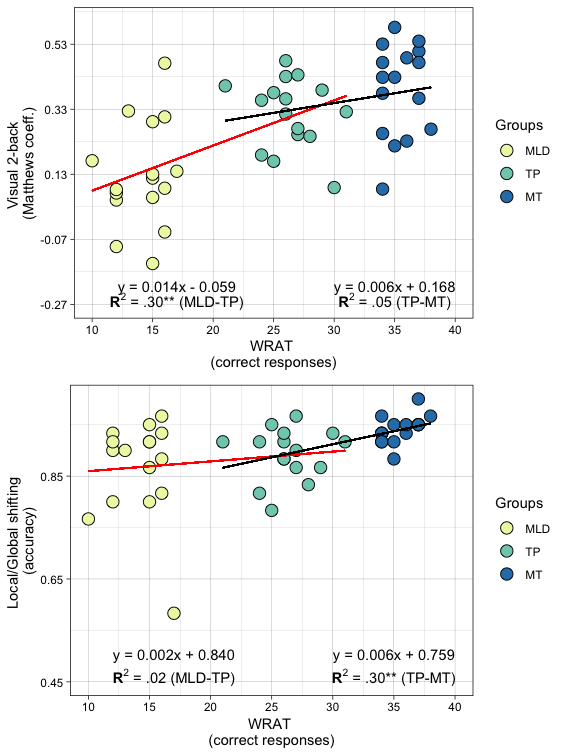


**Fig A. Scatterplots for the correlation between the WRAT Math Computation subtest and accuracy in the visual 2-back task (upper panel) and the local/global task (lower panel).** Red lines show the regression lines considering only the Math Computation scores of the MLD and TP groups, while black lines consider only those of the TP and MT groups. **p < .01
